# Analgesic effect of dexmedetomidine in colorectal cancer patients undergoing laparoscopic surgery

**DOI:** 10.15537/smj.2022.43.10.20220526

**Published:** 2022-10

**Authors:** Ja E. Lee, Hue J. Park, Yoon J. Chung, Hyun J. Ahn, Woo S. Sim, Jin Y. Lee

**Affiliations:** *From the Department of Anesthesiology and Pain Medicine (JE. Lee, Chung, Ahn, Sim, JY. Lee), Samsung Medical Center, School of Medicine, Sungkyunkwan University, and from the Department of Anesthesiology and Pain Medicine (Park), Seoul St. Mary’s hospital, College of Medicine, The Catholic University of Korea, Seoul, Korea.*

**Keywords:** cancer, colon, dexmedetomidine, pain, postoperative

## Abstract

**Objectives:**

To evaluate the analgesic efficacy of intraoperative dexmedetomidine (DEX) for acute postoperative pain in colorectal cancer patients undergoing laparoscopic surgery.

**Methods:**

We retrospectively analyzed data of 190 colorectal cancer patients who had undergone laparoscopic surgery between October 2020 and May 2021 at Samsung Medical Center, Seoul, Korea, with (n=74) or without intraoperative DEX (n=85) administration. The demographic, clinical, anesthetic, and postoperative data were compared.

**Results:**

In total, 159 patients were enrolled. Demographic and clinical data were not different between the groups. The mean arterial pressure (*p*<0.001) and heart rate (*p*<0.001) were lower in the DEX group at the end of surgery and after extubation (*p*=0.003, *p*=0.001). The minimum alveolar concentration of sevoflurane was lower in the DEX group during surgery. At the post-anesthesia care unit (PACU) admission and discharge, pain scores (*p*<0.001, *p*=0.027) and fentanyl consumption (*p*<0.001) were significantly lower in the DEX group. On postoperative days 1-3, pain scores and opioid consumption were not different between the groups. The incidence of postoperative complications was not different between the groups.

**Conclusion:**

Continuous intraoperative DEX administration had an intraoperative analgesic effect as indicated by lower hemodynamic and fentanyl consumption. Furthermore, there was immediate postoperative analgesia as suspected by the lower pain scores and fentanyl dose during the PACU. However, pain scores and opioid consumption after the PACU remained unaffected.

**D**espite remarkable developments in minimally invasive surgical techniques and understanding of pain mechanisms, postoperative pain remains a serious issue.^1^ Surgical tissue and neural damage activate nociceptive neurons, leading to peripheral and central sensitization.^2^ Acute postoperative pain mostly revolves within one week; however, in approximately 10% of all surgeries, acute postoperative pain persists beyond the usual tissue healing time and transits into persistent postoperative pain.^2^ The presence and intensity of intraoperative pain comprise the most important determinants of persistent postoperative pain.^2,3^ Therefore, multimodal analgesic management is needed to attenuate nociceptor sensitization and decrease postoperative pain.^4,5^ Opioids have been considered the gold standard of acute postoperative pain management for decades.^3^ They provide balanced anesthesia and analgesia by blunting the hemodynamic response to surgical stimuli.^6^ However, opioid monotherapy is limited due to adverse events (respiratory depression, oversedation, and rapid development of tolerance).^7^ Therefore, opioid-sparing analgesia is a critical component of acute and chronic pain management to enhance analgesic efficacy and eliminate adverse effects. Large numbers of non-opioids, such as nonsteroidal anti-inflammatory drugs (NSAIDs), acetaminophen, or alpha-2 agonist, have improved analgesic properties.^8^

Dexmedetomidine (DEX), a highly selective and potent agonist of alpha-2 adrenoreceptors, acts on neuropathic pain modulation.^9,10^ It has sedative, sympatholytic, and analgesic effect.^11^ Dexmedetomidine inhibits norepinephrine release from synaptic neurons via alpha-2 receptors in rostral pons and central pain modification by enhancing the inhibitory descending pain pathway.^11,12^ Reportedly, DEX diminishes the catecholamine levels by decreasing the sympathetic nervous system activity and provide hemodynamic stability during surgery.^13^ Dexmedetomidine has been used as a sub-anesthetic and anxiolytic agent; however, of late, DEX has been found to have an antinociceptive effect, thus broadening its usage in acute and chronic pain conditions. Perioperative administration of DEX has been shown to have superior analgesic and opioid-sparing effects in several surgeries.^14-17^ In laparoscopic hysterectomy, intraoperative DEX showed decreased postoperative pain and improved bowel function.^11,18^ In spinal fusion surgery, intraoperative DEX did not decrease postoperative pain and opioid consumption.^11^ Nonetheless, the analgesic efficacy of DEX is still unclear due to different surgical and pain characteristics. In laparoscopic colon surgery, the enhanced recovery after surgery (ERAS) guideline recommends multimodal analgesia using opioids and non-opioids (NSAIDs or paracetamol).^19,20^ Published studies regarding DEX in colorectal surgery focused on cognitive dysfunction, cardiovascular stability, or cerebral protection effect; however, these studies are insufficient to determine the analgesic efficacy of DEX. Therefore, the aim of this syudy was to evaluate the efficacy of intraoperative DEX on postoperative pain in colorectal cancer surgery.

## Methods

We retrospectively examined the electronic medical records of colorectal cancer patients who underwent laparoscopic surgery between October 2020 and May 2021 at Samsung Medical Center, Seoul, Korea. The patients were aged 27-87 years. The inclusion criteria were as follow: I) patients who were diagnosed with malignant tumors in the colon or rectum; and II) who have undergone elective laparoscopic colectomy or anterior resection.^21^ The exclusion criteria were as follow: I) non-elective surgery; II) metastatic cancer surgery; III) open or conversion to open surgery; IV) insufficient follow-up data; V) intensive care unit discharge after surgery; VI) inability to express pain intensity; and VII) usage of any of the analgesics (NSAIDs, acetaminophen, or dexamethasone) during anesthesia.^21^ This study was carried out according to the guidelines of the Declaration of Helsinki. It was approved by the Institutional Review Board of Samsung Medical Center, Seoul, Korea, (SMC 2021-07-089) and registered with the Clinical Research Information Service of the Korea National Institute of Health, (ref: KCT0006382). Since this was a retrospective review of medical records, patient consent was waived.

The patients were not premedicated preoperatively. In the operating room, standard monitoring for anesthesia was carried out, including oxygen saturation, electrocardiography, end-tidal carbon dioxide (CO2), bispectral index (BIS), oxygen reserve index, and non-invasive blood pressure. Anesthesia was induced with 40 mg of 2% lidocaine, 2 mg/kg of 2% propofol, 0.5-1.0 μg/kg of fentanyl, 0.05 mg/kg of midazolam, and 0.6-0.8 mg/kg of rocuronium intravenously. At anesthetic induction, 0.5-1.0 μg/kg/hour of DEX (Precedex^®^, Hospira, Lake Forest, IL, USA) was administered without a bolus dose, and continued until the end of anesthesia. Endotracheal intubation was carried out after approximately 3-5 minutes of mask ventilation and loss of all 4 twitches by train-of-4 stimulation of the ulnar nerve. After endotracheal intubation, anesthesia was maintained with 1.5-2.0 vol% sevoflurane and a bolus injection of 0.5-1.0 μg/kg fentanyl to maintain hemodynamic parameters within 20% of baseline values and BIS at 30-60. The lungs were ventilated with 50% oxygen with air. This was adjusted to maintain an end-tidal CO2 level of 30-40 mmHg. Intraoperative fluid was managed considering blood pressure, heart rate, and hemoglobin level. The temperature was maintained within normal ranges using a warm blanket. All surgeries were carried out by specialized surgeons who followed standardized procedures for colorectal cancer.^22^ At the end of the surgery, patients received intravenous patient-controlled analgesia (PCA) pump (Automed3200^®^, Ace Medical, South Korea), which delivered 10-20 μg/kg of fentanyl in normal saline (100 ml) at a basal infusion rate of 0.5 ml/hour and bolus of 1 ml. At the end of the anesthesia, the patients were administered 4 mg/kg of sugammadex or 0.03 mg/kg of pyridostigmine and 0.002 mg/kg of glycopyrrolate intravenously. After extubation, the patients were transferred to the post-anesthesia care unit (PACU). They received a further bolus of intravenous fentanyl at 0.5 μg/kg when the reported scores on a numeric rating scale (NRS: 0=no pain to 10=absolutely intolerable pain) were greater than 3. After discharge from the PACU, postoperative intravenous opioids (fentanyl, pethidine, or hydromorphone) and oral opioids (oxycodone or tapentadol) were administered in the general ward according to the attending physician’s decision.

The primary endpoints were postoperative pain and opioid consumption. The pain scores were checked at PACU arrival, PACU discharge, and postoperative day (POD) 1, 2, and 3. Opioid consumption was recorded by conversion to fentanyl units throughout POD 1, 2, and 3.^22^ The secondary endpoint was the incidence of postoperative complications.

### Statistical analysis

All data were analyzed using SAS 9.4 (SAS Institute, Cary, NC, USA). Data are described as mean ± standard deviation (SD) or number (n) and precentage (%), as appropriate. Demographic, clinical, and anesthetic data for the 2 groups were compared using a Chi-square test, T-test or Fisher’s exact test. We extracted the mean arterial pressure, heart rate, BIS, oxygen saturation, and oxygen reserve index before intubation (T0), at the start of surgery (T1), at the start of intra-abdominal CO2 insufflation (T2), at deflation of intra-abdominal CO2 (T3), at the end of surgery (T4), and after extubation (T5). The values of minimum alveolar concentration (MAC) of sevoflurane were extracted from T1-T4. These values were compared using a T-test or Wilcoxon rank sum test. In each group, differences in mean arterial pressure, heart rate, BIS, and MAC of sevoflurane values over time were compared using a generalized estimating equation (GEE) analysis. We analyzed the pain scores at PACU arrival and PACU discharge and on POD 1, 2, and 3 at rest and during movement. We analyzed opioid consumption throughout anesthesia, while admitted to the PACU, and on POD 1, 2, and 3, using the Wilcoxon rank sum test. We compared the incidence of postoperative complications using a Chi-square test. The level of statistical significance was set at *p*-value of <0.05.

## Results

Of the enrolled 190 patients, 31 were ruled out due to exclusion criteria. Thus, data were evaluated for 159 patients. Patients were divided into 2 groups based on whether they received DEX infusion intraoperatively; patients who received DEX infusion during anesthesia comprised the DEX group (n=74) and those who did not receive DEX infusion comprised the non-DEX group (n=85). The demographic and clinical data are summarized in Table 1. In the DEX group, the mean dose of infused DEX was 70.6±24.5 μg. The intraoperative fentanyl dose was significantly lower in the DEX group (*p*=0.008). Details regarding intraoperative hemodynamics are shown in Table 2. Compared to the non-DEX group, the mean arterial pressure was significantly lower in the DEX group at T4 (*p*<0.001) and T5 (*p*=0.003) and the heart rate was significantly lower at T3 (*p*<0.001), T4 (*p*<0.001), and T5 (*p*=0.001). Moreover, the BIS (*p*=0.016) and oxygen reserve index (*p*=0.030) were significantly lower in the DEX group at T4; however, no between-group differences were observed at other time points. Oxygen saturation was not different between the groups. The MAC of sevoflurane was significantly lower in the DEX group at T2 (*p*=0.002) and T3 (*p*<0.001). The GEE analysis revealed significant between-group differences in the mean arterial pressure (*p*<0.001) and heart rate (*p*<0.001) over time (from T0-5). There were no significant differences in the BIS and MAC of sevoflurane over time between both groups. Table 3 shows the postoperative pain scores. We found that the NRS scores at PACU admission (*p*<0.001) and discharge (*p*=0.027) were significantly lower in the DEX group; however, the NRS scores throughout POD 1-3 were not significantly different between the groups. Table 4 shows the postoperative data. The fentanyl dose during PACU admission was significantly lower in the DEX group (*p*<0.001). From POD 1-3, the fentanyl dose was not different between the groups. Postoperatively, the discontinuation day of PCA, time to pass flatus, and length of hospital stay were not different between the groups. Table 5 shows the postoperative complications. The incidence was not different between the groups.

**Table 1:** - Demographic and clinical data.

Variables	All patients (N=159)	DEX group (n=74)	Non-DEX group (n=85)	*P*-values
Age (year)	61.0±11.6	60.5±10.1	61.5±12.7	0.424
Gender (male/female), n	70/89	28/46	42/43	0.143
Body mass index (kg/m^2^)	23.9±3.2	24.0±3.3	23.8±3.1	0.585
ASA status (I/II/III), n	60/94/5	30/42/2	30/52/3	0.797
***Diagnosis, n (%)***				
Colon cancer	111 (69.8)	51 (68.9)	60 (70.6)	0.819
Rectal cancer	48 (30.2)	23 (31.1)	25 (29.4)	
***Surgery type, n (%)***				
Colectomy	67 (42.1)	34 (45.9)	33 (38.8)	0.364
Anterior resection	92 (57.9)	40 (54.1)	52 (61.2)	
Pathological stage (0/1/2/3/4), n	17/19/33/20/70	9/8/14/12/31	8/11/19/8/39	0.689
Maximum tumor size >4 cm, n (%)	41 (25.8)	20 (27.0)	21 (24.7)	0.739
Anesthesia time (minute)	174.9±40.7	174.8±41.1	175.0±40.7	0.992
Operation time (minute)	130.6±38.5	130.6±38.8	130.7±38.5	0.984
Intraoperative fentanyl dose (μg)	64.6±27.6	57.6±20.7	70.7±31.4	0.008
Rocuronium dose (mg)	72.7±13.0	72.8±12.8	72.6±13.4	0.877

Values are presented as mean ± standard deviation (SD). DEX: dexmedetomidine, ASA: American Society of Anesthesiologists

**Table 2:** - Intraoperative hemodynamics over time.

Variables	All patients (N=159)	DEX group (n=74)	Non-DEX group (n=85)	*P*-values
***Mean arterial pressure***				
T0	94.0±13.0	93.5±14.5	94.4±11.5	>0.999
T1	91.6±18.9	89.1±21.8	93.7±15.8	0.376
T2	104.0±20.1	102.1±19.0	105.5±21.0	>0.999
T3	83.3±12.9	80.6±13.1	85.6±12.4	0.094
T4	82.3±16.6	74.0±12.6	89.5±16.4	<0.001
T5	96.9±16.1	92.3±14.0	100.8±16.9	0.003
***Heart rate***				
T0	75.4±12.9	75.1±12.2	75.7±13.5	>0.999
T1	81.9±15.7	81.1±17.0	82.7±14.5	>0.999
T2	83.7±16.5	82.7±17.5	84.7±15.5	>0.999
T3	68.6±12.0	63.5±10.6	72.9±11.3	<0.001
T4	66.6±12.1	60.3±10.3	72.0±10.9	<0.001
T5	82.5±13.9	76.8±11.6	87.3±13.9	0.001
***Bispectral index***				
T0	94.3±3.8	94.6±4.0	94.1±3.6	0.379
T1	39.2±9.3	39.5±10.4	38.9±8.2	>0.999
T2	37.8±8.0	37.4±8.1	38.1±8.0	>0.999
T3	40.5±7.1	40.7±7.4	40.3±6.9	>0.999
T4	51.5±9.9	49.0±10.1	53.7±9.1	0.016
T5	80.7±7.7	81.1±8.6	80.3±6.8	>0.999
***Oxygen saturation (%)***				
T0	99.3±1.0	99.3±0.9	99.2±1.1	>0.999
T1	99.9±0.4	99.8±0.5	99.9±0.4	>0.999
T2	99.8±0.5	99.9±0.5	99.8±0.5	>0.999
T3	99.9±0.4	99.9±0.4	99.9±0.3	>0.999
T4	99.9±0.3	99.9±0.4	100.0±0.2	>0.999
T5	99.9±0.6	99.8±0.7	99.9±0.4	>0.999
***Oxygen reserve index***				
T0	0.0±0.1	0.0±0.1	0.0±0.1	>0.999
T1	0.4±0.2	0.4±0.2	0.4±0.2	0.183
T2	0.4±0.2	0.4±0.2	0.4±0.2	0.887
T3	0.4±0.3	0.4±0.3	0.4±0.3	>0.999
T4	0.6±0.3	0.5±0.3	0.6±0.3	0.030
T5	0.5±0.5	0.5±0.7	0.5±0.3	>0.999
***MAC of sevoflurane***				
T1	1.1±0.3	1.0±0.3	1.1±0.2	0.051
T2	1.2±0.3	1.0±0.3	1.2±0.3	0.002
T3	0.9±0.2	0.9±0.2	1.0±0.2	<0.001
T4	0.4±0.2	0.4±0.2	0.5±0.2	0.843

Values are presented as mean ± standard deviation (SD). DEX: dexmedetomidine, MAC: minimum alveolar concentration, T0: before intubation, T1: start of surgery, T2: insufflation of intra-abdominal CO2, T3: deflation of intra-abdominal CO2, T4: end of surgery, T5: after extubation

**Table 3:** - Postoperative pain scores.

Postoperative pain (NRS)	All patients (n=159)	DEX group (n=74)	Non-DEX group (n=85)	*P*-values
***PACU***				
At admission	6.3±2.2	5.6±2.2	6.9±2.0	<0.001
At discharge	2.8±1.0	2.7±1.1	2.8±0.6	0.027
***POD 1***				
Rest	2.9±0.3	2.9±0.4	2.9±0.3	>0.999
Movement	4.8±1.7	5.0±1.7	4.7±1.7	>0.999
***POD 2***				
Rest	3.0±0.2	3.0±0.2	3.0±0.2	>0.999
Movement	4.3±1.6	4.4±1.6	4.2±1.5	>0.999
***POD 3***				
Rest	2.9±0.3	2.9±0.3	2.9±0.3	>0.999
Movement	3.8±1.4	3.9±1.5	3.5±1.2	>0.999

Values are presented as mean ± standard deviation (SD). DEX: dexmedetomidine, NRS: numeric rating scale, PACU: post-anesthesia care unit, POD: postoperative day

**Table 4:** - Postoperative data.

Variables	All patients (N=159)	DEX group (n=74)	Non-DEX group (n=85)	*P*-values
Fentanyl dose for PCA (μg)	1199.4±200.2	1210.8±197.0	1189.4±203.6	0.499
Fentanyl dose for PACU (μg)	39.5±28.9	27.9±23.1	49.6±29.8	<0.001
***Fentanyl dose after surgery (μg)***				
POD 1	72.9±80.5	73.3±77.5	72.5±83.5	>0.999
POD 2	140.3±105.3	123.5±105.6	154.9±103.4	0.244
POD 3	168.9±160.9	184.7±195.3	155.2±123.0	>0.999
Discontinuation day of PCA (POD)	2.4±0.9	2.3±1.0	2.5±0.8	0.080
Time to pass flatus (POD)	2.9±1.0	3.0±1.0	2.8±1.0	0.314
Length of hospital stay (POD)	6.3±1.2	6.2±1.3	6.4±1.1	0.076

Values are presented as mean ± standard deviation (SD). DEX: dexmedetomidine, PCA: patient-controlled analgesia, PACU: post-anesthesia care unit, POD: postoperative day

**Table 5:** - Postoperative complications.

Complications	All patients (N=159)	DEX group (n=74)	Non-DEX group (n=85)	*P*-values
Nausea/vomiting	35 (20.0)	16 (21.6)	19 (22.4)	0.912
Itching	3 (1.9)	1 (1.4)	2 (2.4)	>0.999
Dizziness	9 (5.7)	4 (5.4)	5 (5.9)	>0.999
Headache	6 (3.8)	3 (4.1)	3 (3.5)	>0.999

Values are presented as a number and precentage (%). DEX: dexmedetomidine

## Discussion

In this study, we aimed to evaluate the analgesic effectiveness of intraoperative DEX in colorectal cancer patients undergoing laparoscopic surgery. Dexmedetomidine had intraoperative analgesia as indicated by the lower mean arterial pressure, heart rate, and MAC of sevoflurane, as well as the reduced fentanyl consumption by 18.5%. Postoperatively, it induced analgesia based on lowered pain scores and a 43.8% reduction in fentanyl consumption during PACU admission. After PACU discharge, pain scores and opioid consumptions were not different on POD 1, 2, and 3 between both groups. Notably, the incidence of postoperative nausea/vomiting, dizziness, itching, and headache was not different between both groups. Continuous DEX infusion reduced intraoperative pain, but not postoperative pain. We hypothesize that although intraoperative DEX has an analgesic effect, the short half-time of DEX or dose of DEX is insufficient to sustain the analgesic effect for postoperative pain.^23^ Therefore, continuous multimodal analgesia is required to achieve prolonged analgesia after surgery.^8^ Furthermore, postoperative pain after colorectal surgery has a mixed character (nociceptive, visceral, and neuropathic pain); since the pelvic cavity is a limited space, there is an increased risk of damage to the pelvic nerve plexus during surgery.^22,24^ These multiple sources of pain may induce more severe pain compared to other laparoscopic surgeries; therefore, intraoperative DEX infusion induce postoperative analgesia not lasting over 24 hours.

The ERAS recommendations included the combined use of non-opioid analgesics with opioids.^5^ However, the application of various analgesic protocols has shown different results regarding postoperative pain management.^7,19,20,25^ Dexmedetomidine has previously been administered intravenously for sedation and analgesia during critical illness and surgical procedures.^26^ It induces analgesia by releasing centrally acting enkephalin-like substance, inhibits the release of substance P at the level of the dorsal root neuron, and prevents norepinephrine release at nerve endings.^11^ Dexmedetomidine has anti-inflammatory properties indicated by the reduced secretion of inflammatory factors and decreased inhibition of immunity, leading to reduced opioid consumption.^27^ It was shown to have a rapid distribution corresponding to a half-time of 6 minutes, followed by a 2-hour terminal half-time and 4 hours context sensitive half-time after a prolonged 8 hours infusion.^12,28^ The adverse effects of DEX include hypotension, hypertension, nausea, bradycardia, atrial fibrillation, and hypoxia.^28^ When DEX is infused at a higher bolus dose, it triggers tachycardia and hypertension. Therefore, further studies are warranted to determine the optimal dose of DEX. Several studies have reported improved analgesia when DEX was used as an adjuvant to anesthetic agents via various routes (intravenous, nasal, oral, intramuscular, intraperitoneal, and interfascial plane).^12,28^ In laparoscopic colon cancer surgery, 0.5-1.5 μg/kg DEX was administered with ropivacaine for transversus abdominis plane block during anesthesia, and it showed improved postoperative pain compared with a placebo group.^29^ Intravenous DEX combined with sufentanil showed significantly decreased pain score until post-operative 48 hours after colon cancer surgery.^30^ Intravenous analgesic regimens of DEX administration for adults recommend 0.25-0.5 μg/kg for the loading bolus and 0.2-1.0 μg/kg/hour for continuous injection.^12,31^ A comparison of continuous injection of DEX with or without a loading dose showed no differences in 24 hours postoperative opioid consumption after abdominal surgery.^12,32^ In our study, we infused DEX corresponding to 0.5-1.0 μg/kg/hour continuously and stopped the infusion at the end of anesthesia; we observed that the analgesic effect was significant until PACU discharge. Considering the sedative effects of DEX and the importance of early ambulation after surgery, other analgesics or interventions are required to prolong analgesia postoperatively.

### Study limitations

First, we did not enroll postoperatively prescribed non-opioid analgesics (acetaminophen, NSAIDs, or tramadol) into opioid equivalents due to conversion difficulties, even though this could have affected postoperative pain. Second, we did not measure the daily required dose of PCA, which may reflect daily pain intensity. Third, this retrospective study had a small sample size. Further studies with larger sample sizes are needed to investigate prolonged analgesia using other non-opioid analgesics for laparoscopic colorectal surgery.

In conclusion, intraoperative continuous DEX administration showed analgesic effects intraoperatively and immediately after surgery but not during the postoperative period. Further prospective trials are needed to investigate analgesic prolongation with different doses or administration routes of DEX and with the combined use of other adjuvants or interventions.
